# Placenta Creta: A Spectrum of Lesions Associated with Shallow Placental Implantation

**DOI:** 10.1155/2020/4230451

**Published:** 2020-11-24

**Authors:** Jerzy Stanek

**Affiliations:** Division of Pathology, Cincinnati Children's Hospital Medical Center, 3333 Burnet Avenue, Cincinnati, OH 45229, USA

## Abstract

**Background:**

On placental histology, placenta creta (PC) ranges from clinical placenta percreta through placenta increta and accreta (clinical and occult) to myometrial fibers with intervening decidua. This retrospective study aimed to investigate the clinicopathologic correlations of these lesions.

**Methods:**

A total of 169 recent consecutive cases with PC (group 1) were compared with 1661 cases without PC examined during the same period (group 2). The frequencies of 25 independent clinical and 40 placental phenotypes were statistically compared between the groups using chi-square test or analysis of variance where appropriate.

**Results:**

Group 1 placentas, as compared with group 2 placentas, were statistically significantly (*p* < 0.05) associated with caesarean sections (11.2% vs. 7.5%), antepartum hemorrhage (17.7% vs 11.6.%), gestational hypertension (11.2% vs 4.3%), preeclampsia (11.8% vs 2.6%), complicated third stage of labor (18.9% vs 6.4%), villous infarction (14.2% vs 8.9%), chronic hypoxic patterns of placental injury, particularly the uterine pattern (14.8%, vs 9.6%), massive perivillous fibrin deposition (9.5% vs 5.3%), chorionic disc chorionic microcysts (21.9% vs 15.9%), clusters of maternal floor multinucleate trophoblasts (27.8% vs 21.2%), excessive trophoblasts of chorionic disc (24.3% vs 17.3%), segmental fetal vascular malperfusion (27.8% vs 19.9%), and fetal vascular ectasia (26.2% vs 15.2%).

**Conclusion:**

Because of the association of PC with gestational hypertensive diseases, acute and chronic placental hypoxic lesions, increased extravillous trophoblasts in the chorionic disc, chorionic microcysts, and maternal floor trophoblastic giant cells, PC should be regarded as a lesion of abnormal placental implantation and abnormal trophoblast invasion rather than decidual deficiency only.

## 1. Introduction

The placenta creta (PC) spectrum is an important contributor to maternal morbidity. Several excellent reviews have discussed the clinical risk factors for PC, the most important being placenta previa, uterine scars, and previous abnormal placental separation, as well as signs and symptoms, staging (accreta, increta, and percreta), management, and clinicopathological correlation [1–7]. The incidence rate of histological placenta accreta in women with placenta praevia was 40.49% [8].

Ultrasound can be used to make a prenatal suggestion of PC spectrum disorders [1, 8]. It is the primary method for diagnosing morbidly adherent placenta, with colour Doppler providing the best diagnostic performance, but magnetic resonance imaging should be considered when planning a resection.

The risk for major hemorrhagic morbidity after a prior pathologically diagnosed PC depends on the clinical context. Preparation for major blood loss is indicated after a prior pregnancy complicated by hemorrhage or with treatment of retained placenta with histological placenta accreta [9].

Occult PC is more common than clinical PC, but its true incidence is probably higher (sampling dependence) when more than the usual number of placental sections are obtained. In addition to clinical risk factors, basal plater myometrial fibres (BPMF) are more common when gross disruption of the basal plate is observed and sections are taken from those areas, including *en face* sections [10].

Clinically symptomatic cases of PC are usually associated with known risk factors or complications whereas occult PC might not be, [6, 10] and histological PC may occur with or without clinical suspicion of the condition. BPMF on placental histology are associated with an increased risk of morbidly adherent placenta in a subsequent pregnancy [11].

The roles of genetics, local hormonal factors, and the trophoblast itself have yet to be established [3]. BPMF with or without intervening decidua are the earliest clinically asymptomatic lesions of abnormal placentation. As it is tempting to include BPMF into the lesions of shallow placental implantation, this analysis aimed to investigate the clinicopathologic correlations of the spectrum of PC including all early/subclinical and clinical varieties and thereby to complement previous reports on histological features of shallow placental implantation in preeclampsia [12], mass-forming congenital anomalies without chromosomal abnormalities or heart malformations [13], and maternal diabetes mellitus complicated by hypertensive condition of pregnancy [14].

## 2. Methods

The study was approved by the institutional review board (IRB #2016-7942). Placental examination was performed according to the recommendations and nomenclature of the Amsterdam Consensus Conference [15] except for some abnormal placental phenotypes that were previously defined elsewhere [16–24]. The individual placentas were examined by pediatric pathologists of the Division of Pathology (9), but all of them were re-examined by the author whose final results were included in the analysis. All gross lesions and margins of disruption of the maternal floor were sampled, the latter shaved, and at least 2 sections of the membrane rolls and the umbilical cord and 2 paracentral sections of grossly unremarkable placenta were also obtained. Formalin-fixed and paraffin-embedded sections were stained with haematoxylin and eosin (H&E) and were reviewed by the author.

Of 1830 consecutive placentas >19 of weeks gestation, examined by the author in years 2006 till 2019, 9.2% (169 cases) showed lesions of the PC spectrum (group 1). Cases with BPMF with intervening decidua and clinical and occult placenta accreta cases are included as it appears that BPMF also correlate with the increased amount of extravillous trophoblasts and other features of shallow placental implantation.

**Figure 1:**
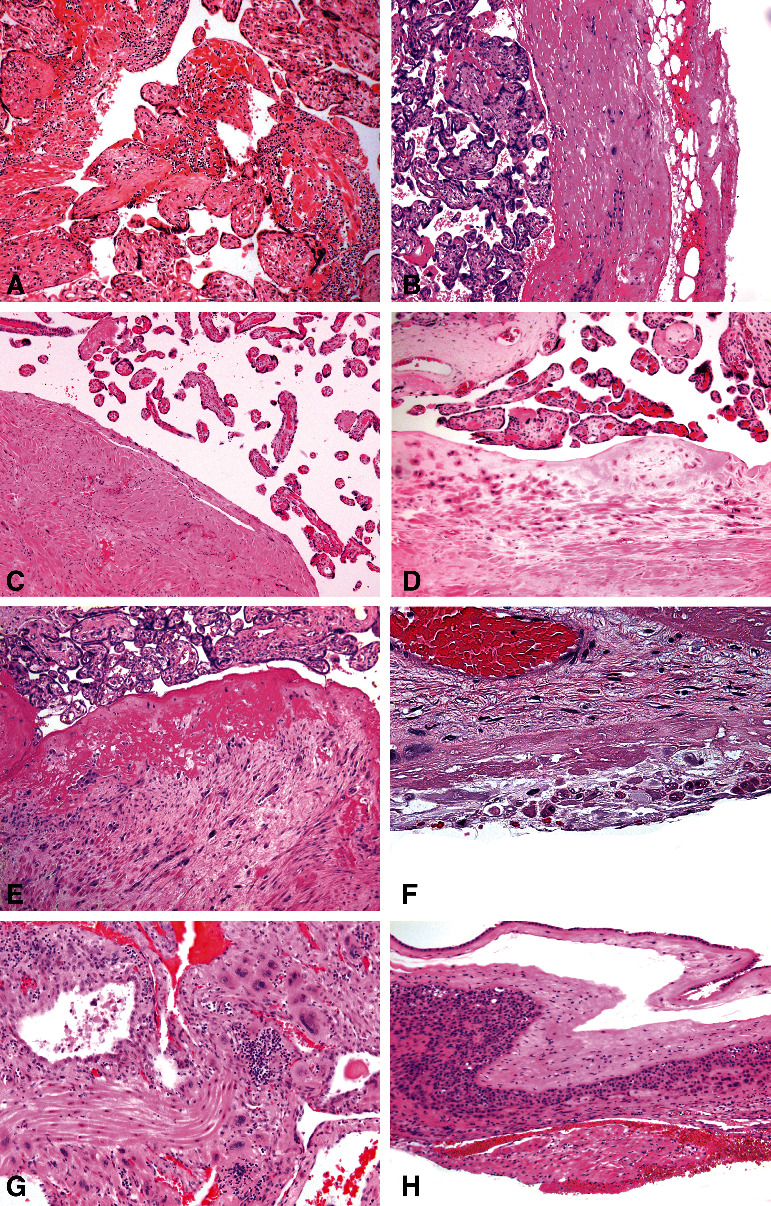
Placenta creta spectrum (haematoxylin and eosin, original objective magnifications are given in parentheses). (a) Placenta increta: myometrial fibers are intermingled with chorionic villi (20x). (b) Placenta percreta: placental tissue is separated from the subperitoneal fat only by fibrous tissue (10x). (c) Symptomatic placenta accreta: myometrial tissue in direct contact with the intervillous space (10x). (d) Occult placenta accreta: extravillous trophoblasts with underlying myometrial fibers in direct contact with the intervillous space (10x). (e) Occult placenta accreta: Rohr fibrinoid with an underlying mix of myometrial fibers and extravillous trophoblast (10x). (f) Basal plate myometrial fibers: Rohr fibrinoid with underlying decidua, Nitabush fibrinoid, and myometrial fibers (40x). (g) Myometrial fibers in a shaved section from a margin of disruption of the maternal floor (20x). (h) Placental membranes with adjacent myometrial fibers (chorion leave accreta) (10x).

Therefore, the inclusion criteria were findings of smooth muscle fascicles attached to maternal floor and/or placental membranes [25] with intervening decidua, myometrial fibers adjacent to the maternal floor extravillous trophoblasts or Rohr fibrinoid without intervening decidua, or myometrial fibers in direct contact with the anchoring chorionic villi, as well as cases of clinical placenta accreta, increta and percreta (Figure 1). All group 1 placentas met the above inclusion criteria. Apart from gestational age <20 weeks, there were no exclusion criteria. Cases in group 1 were compared to the remaining 1661 cases without PC (group 2) which were examined in the same time period. Smooth muscle actin immunostain stain was performed when eosinophilic wisps were seen at the basal plate or membrane roll to highlight muscle fibers. The frequencies of 25 independent clinical and 40 placental phenotypes were statistically compared between the groups through analysis of variance or chi-square, where appropriate.

## 3. Results

In group 1, symptomatic PC was seen in 35 cases (20.7%): 27 cases of symptomatic placenta accreta, 3 cases of placenta increta, and 5 cases of placenta percreta, but occult PC with or without intervening decidua was by far most common (134 cases, 79.3%), and the latter including also 2 cases of occult chorion leave accreta [25].

PC was more common in placentas from caesarean sections which was performed in 56% of the cases in group 1 and in 45% of the cases in group 2. Antepartum hemorrhage, gestational hypertension, preeclampsia and complicated third stage of labor (prolonged, postpartum blood loss), and manual extraction of placenta were also more common in group 1 (Table 1).

**Figure 2:**
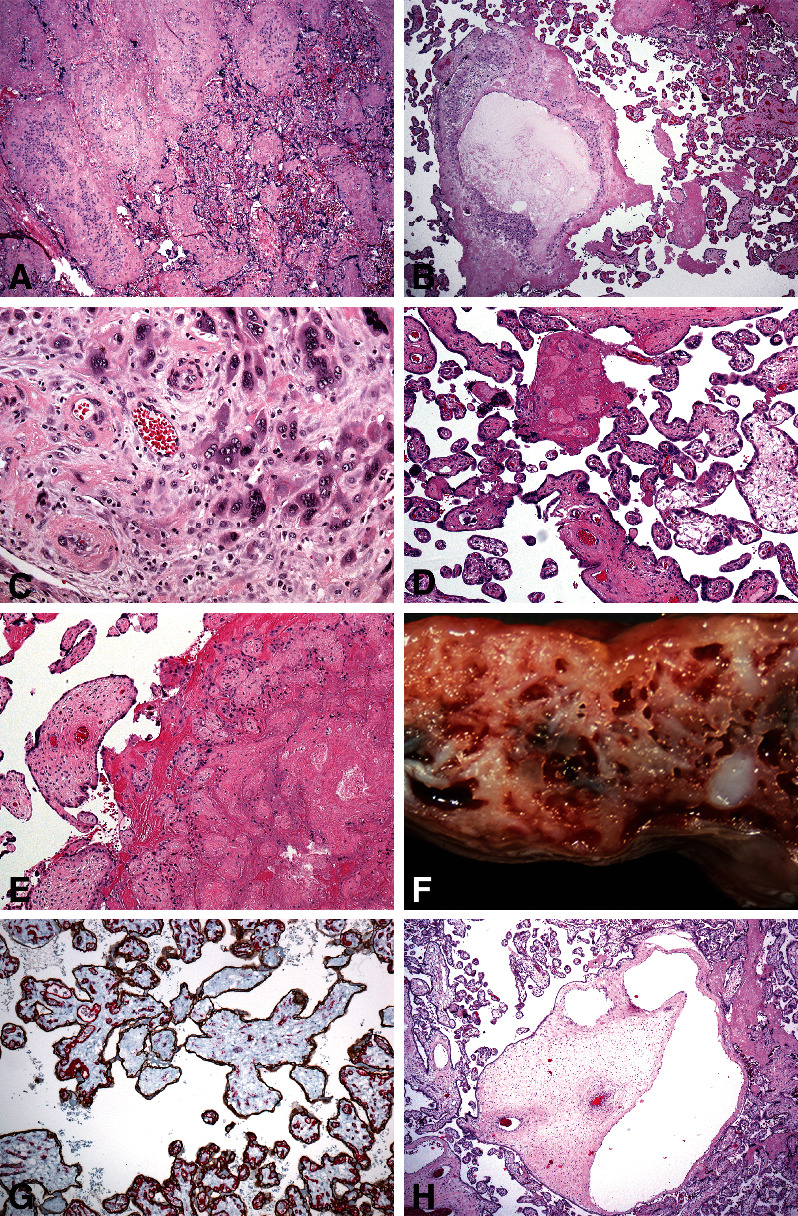
Lesions of shallow placental implantation (a–c), hypoxic lesions (d–f), and fetal vascular malperfusion lesions (g, h) statistically significant more common in group 1 (original objective magnifications are given in parentheses). (a) Confluent cell islands (excessive extravillous trophoblasts) (haematoxylin eosin, 4x). (b) Chorionic microcysts (haematoxylin eosin, 4x). (c) A cluster of multinucleate trophoblasts and hypertrophic decidual arteriopathy in maternal floor (haematoxylin eosin, 40x). (d) Uterine chronic hypoxic pattern at 34 weeks gestation (haematoxylin eosin, 10x). (e) Villous infarction (haematoxylin eosin, 10x). (f) Massive perivillous fibrinoid deposition (dissecting microscopy). (g) Incipient segmental villous hypovascularity (E cadherin (brown)/CD34 (red) immunostain, 10x). (h) Fetal stem vascular ectasia, global fetal vascular malperfusion (haematoxylin and eosin, 4x).

Of placental phenotypes (Table 2), villous infarction, chronic hypoxic patterns of placental injury, particularly the uterine pattern, massive perivillous fibrin deposition, chorionic disc chorionic microcysts, clusters of maternal floor multinucleate trophoblasts, excessive trophoblasts of chorionic disc, segmental fetal villous malperfusion (segmental avascularity, hypovascularity, and/or endothelial fragmentation by CD34 immunohistochemistry) [23], and fetal vascular ectasia were more common in group 1 (Figure 2).

## 4. Discussion

The incidence rate of PC in all studied placentas was 9.2% (Table 1). This is much higher than the 2-3% incidence rate of PC in the general population [1]. However, along with clinical PC, our material includes also occult placenta accreta and basal plate myometrial fibers with intervening decidua (Figure 1). Our 1.9% prevalence of clinical PC is therefore closer to the 2-3% incidence in general population. This study analyzed also other placental variables aside from PC in order to further clarify the pathophysiology of abnormal placental adherence and not merely to repeat previous epidemiological studies of the PC spectrum. It is not surprising that obvious associations with antepartum bleeding, more common deliveries by cesarean section, and complicated third stage of labour in Group 1 were confirmed. It is possible that the higher percentage of caesarean sections in group 1 (Table 1) resulted in more common presence of asymptomatic PC due to mechanical reason (i.e., due to manual separation of placentas during cesarean section) and not due to abnormal placental implantation, but this would require further studies.

As subclinical and clinical manifestations of abnormal placental implantation may have the same pathophysiology [10, 26] and tendency to recur in subsequent pregnancies, [9, 11] this retrospective analysis aimed at expanding our clinicoplacental studies to include the whole spectrum of PC lesions. The current opinion is that decidual deficiency rather than a primarily abnormally invasive trophoblast is most important, [5] but increased numbers of implantation-site intermediate trophoblasts were reported in hysterectomy specimens in cases of PC [27] and occult placenta accreta in nonhysterectomy placentas, [16] which would indicate that occult PC has similar histological characteristics as clinical PC and may share the same pathogenesis (decidual deficiency, abnormal trophoblast/decidua interaction, and/or hypoxia).

Several experimental studies on trophoblast proliferation and invasion in PC exist: [28] excessive protease activity, [25] decidual-trophoblastic antagonism at molecular level, [29] autocrine/paracrine actions of trophoblast-derived insulin growth factor-II in binding insulin growth factor binding protein-1 and as an integrin ligand, [30] vital kinases in mTOR pathways, [31] inflammatory cytokine-enhanced matrix metalloproteinase 9, [32] and laminin binding proteins, [33] to mention a few. However, the above findings are difficult to interpret and apply in everyday clinical practice.

Routine examination of placentas may also provide information on the amount of extravillous trophoblasts: the increased extravillous trophoblast layer in placental membranes with or without chorionic microcyst formation, [20] increased number of cell islands in the chorionic disc with or without chorionic microcysts, [21, 22] and clusters of multinucleate trophoblasts at the maternal flood [34, 35]. The latter association may be indicative of poor placental implantation [36] or poor placental perfusion. [37] Based on these studies, a concept of peculiar histology of shallow placentation emerged, which was observed mostly in preeclampsia, in association with placental hypoxic lesions [12] and fetal growth restriction [38]. This analysis showed more common gestational hypertension and preeclampsia in group 1 (Table 1). However, on routine placental examination, the pattern was not uniquely associated in association with PC, being seen in other clinical situations. The PC spectrum may be thus discussed in terms of abnormal trophoblast attachment and not necessarily invasion [39]. Therefore, for the purpose of this analysis, cases of clinical PC, occult placenta accreta, and BPMF with intervening decidua were pooled together.

This analysis showed an overlap complex of chronic developmental and acute hypoxic placental lesions, fetal vascular malperfusion lesions, and lesions of shallow placental implantation in PC, as in preeclamptic placentas, [12] suggestive of similar pathophysiology reflecting maldistribution of the extravillous trophoblasts, with a large part remaining in the delivered placenta and a smaller part at the placental implantation site in the uterus, without necessarily changing the absolute numbers of extravillous trophoblasts. This also suggests that PC seen more commonly in placentas from caesarean section is unlikely due to purely mechanical reasons, but rather it is pathogenetically related to shallow placental implantation.

The process may be explained by the findings of others on the relationship of extravillous trophoblast invasion and remodeling of spiral arteries resulting in a failure of the second wave of trophoblast invasion in spontaneous abortion and early-onset fetal growth restriction with or without preeclampsia related to PC [16]. Impaired trophoblast invasion can be due to a premature increase in placental oxygen secondary to premature onset of maternal blood flow towards the placenta in the first trimester. Mechanisms such as reduced proliferation, enhanced apoptosis, or even increased fusion of extravillous trophoblasts may be responsible for the reduction of numbers of invasive trophoblasts in the myometrium [40]. Whether the dysregulation of trophoblast invasion is caused by intrinsic (trophoblastic) or environmental (decidual and arterial) factors remains to be elucidated [41, 42]. In the studied placentas, the features of shallow placental implantation were more common than the hypertensive conditions of pregnancy in group 1. Therefore, this cannot be explained solely by pure association with preeclampsia. This study also confirmed the findings of a previous analysis that PC is commonly seen with placental infarctions and chronic hypoxic patterns of placental injury, [16] similar in early-onset preeclampsia [12].

The limitations of the study are its retrospective nature, the fact that the author was not blinded to the clinical information on the patients, and no control group was included. Only a comparative group was studied as no normal placentas were reviewed. Therefore, more structured research would be indicated.

In summary, although the least common, PC should be included into the spectrum of lesions of shallow placental implantation because of its association with hypertensive conditions of pregnancy, placental lesions of maternal vascular malperfusion, and other histological lesions of shallow placental implantation.

## Figures and Tables

**Table 1:** Clinical phenotypes.

	Group 1: placenta creta	Group 2: no placenta creta	*F* or Yates chi-square	*p* < 0.05
Number of cases	169	1661		
Gestational hypertension	19 (11.2%)	72 (4.3%)	15.5	0.001
Preeclampsia	20 (11.8%)	43 (2.6%)	39.4	0.001
Chronic hypertension	5 (3.0%)	47 (2.8%)		
Gestational age (weeks, average ± standard deviation)	32.8 ± 6.6	32.3 ± 7.4		
Maternal diabetes mellitus	9 (5.3%)	114 (6.9%)		
Oligohydramnios	18 (10.6%)	153 (9.2%)		
Polyhydramnios	16 (9.5%)	93 (5.6%)		
Antepartum hemorrhage	30 (17.7%)	193 (11.6%)	5.4	0.020
Abnormal fetal heart rate tracing^a^	26 (15.4%)	331 (19.9%)		
Abnormal umbilical artery Doppler	12 (7.1%)	242 (14.6%)		
Induction of labor	23 (13.6%)	309 (18.6%)		
Placenta previa	3 (1.8%)	11 (0.7%)		
Cesarean section	95 (56.2%)	750 (45.1%)	7.6	0.060
Postcesarean hysterectomy	4 (1.7%)	1 (0.7%)		
Multiple pregnancy	16 (9.5%)	127 (7.6%)		
Neonatal mortality	21 (12.4%)	212 (12.8%)		
Nonmacerated stillbirth	9 (5.3%)	79 (4.6%)		
Macerated stillbirth	22 (13.0%)	275 (16.6%)		
Fetal growth restriction^b^	34 (20.1%)	301 (18.1%)		
Umbilical cord compromise^c^	16 (9.5%)	122 (7.3%)		
Congenital malformations	39 (23.1%)	335 (20.1%)		
Abnormal 3^rd^ stage of labor (prolonged, hemorrhage)	32 (18.9%)	107 (6.4%)	34.1	0.001′
Manual extraction of placenta (not during hysterectomy)	20 (11.8%)	19 (1.1%)	84.0	0.001

^a^Abnormal nonstress test and/or abnormal contraction stress test and/or abnormal intrapartum cardiotocography (prolonged bradycardia and/or prolonged tachycardia and or decrease of fetal heart rate variability and/or late decelerations), ^b^birth weight <10 centile, ^c^variable decelerations, encirclement, true knot, or prolaps.

**Table 2:** Placental phenotypes.

	Group 1: placenta creta	Group 2: no placenta creta	Chi-square or *F*	*p* < 0.05
Number of cases	169	1661		
Placental weight (grams ±standard deviation)	372.8 ± 183.4	361.2 ± 198.1		
*Inflammatory lesions*				
Chronic villitis of unknown etiology	16 (9.5%)	216 (13.0%)		
Plasma cell deciduitis	9 (5.3%)	81 (4.9%)		
*Hypoxic lesions/patterns*				
*Acute*				
Meconium (histological)	65 (38.5%)	710 (42.7)		
Villous infarction (>5% of placental parenchyma)	24 (14.2%)	148 (8.9%)	5.0	0.025
*Chronic*				
Erythroblastosis of fetal blood	32 (18.9%)	265 (15.9%)		
Hyaline necrosis, including atherosis of spiral arterioles	11 (6.5%)	81 (4.9%)		
Patterns of chronic hypoxic placental injury	49 (29.0%)	334 (20.1%)	7.3	0.007
Preuterine	9 (5.3%)	84 (5.1%)		
Uterine	25 (14.8%)	159 (9.6%)	4.6	0.032
Postuterine	15 (8.9%)	91 (5.5%)		
Retroplacental hematoma	9 (5.3%)	108 (6.5%)		
Intervillous thrombus	37 (21.9%)	294 (17.7%)		
*Shallow placental implantation*				
Membrane chorionic microcysts	23 (13.6%)	203 (12.2%)		
Chorionic disc extravillous trophoblast microcysts	37 (21.9%)	264 (15.9%)	4.0	0.045
Maternal floor multinucleate trophoblast giant cells	47 (27.8%)	352 (21.2%)	3.9	0.047
Excessive extravillous trophoblasts in chorionic disc	41 (24.3%)	288 (17.3%)	5.0	0.026
*Fetal vascular malperfusion*				
Segmental fetal vascular malperfusion	47 (27.4%)	330 (19.9%)	5.9	0.015
Segmental stromal vascular karyorrhexis	14 (8.3%)	100 (6.0%)		
Segmental villous mineralization	12 (7.1%)	146 (8.8%)		
Fetal vascular ectasia	45 (26.2%)	253 (15.2%)	14.6	0.001
Stem vessel obliteration	16 (9.5%)	135 (8.1%)		
Intramural fibrin deposition	7 (4.1%)	124 (7.5%)		
*Other*				
Massive perivillous fibrin deposition (>30% of placental parenchyma)	16 (9.5%)	88 (5.3%)	5.0	0.026
Choriodecidual hemosiderosis	10 (5.9%)	115 (6.9%)		
Villous edema	9 (5.3%)	141 (8.5%)		
Marginal insertion of umbilical cord	18 (10.6%)	112 (6.7%)		
Velamentous insertion of umbilical cord	7 (4.1%)	52 (3.1%)		
Other umbilical cord abnormalities	30 (17.7%)	278 (16.7%)		

## Data Availability

The data used to support the findings of this study are available upon request.
